# Vacuum joints of CuCrZr alloy for high-heat-load photon absorber

**DOI:** 10.1107/S160057752101273X

**Published:** 2022-02-07

**Authors:** Yongjun Li, Limin Jin, Wanqian Zhu, Song Xue, Min Zhang, Shuai Wu

**Affiliations:** a Shanghai Synchrotron Radiation Facility, Zhangjiang Laboratory, Shanghai Advanced Research Institute, Chinese Academy of Sciences, Shanghai 201204, People’s Republic of China

**Keywords:** CuCrZr, tungsten inert gas welding (TIG), electron beam welding (EBW), finite-element analysis (FEA), high-heat-load photon absorber

## Abstract

A photon absorber, as a critical component of a synchrotron front-end, is mainly used to handle high-heat-load synchrotron radiation. It is mostly made of dispersion strengthened copper or CuCrZr which can retain high performance at elevated temperatures. Joining processes for vacuum, including tungsten inert gas welding (TIG) and electron beam welding (EBW), are novel ways to make a long photon absorber from two short ones and reduce power density. The mechanical properties of TIG joints and EBW joints of CuCrZr to the same material are obtained by tensile tests at 20°C, 100°C, 200°C, 300°C and 400°C. Testing results indicate that the tensile strength and yield strength of both vacuum joints decline as temperature increases. Compared with TIG joints, EBW joints have higher strength, better ductility and a more stable performance. An engineering conservative acceptance criteria of the vacuum joints is created by the polynomial fitting method. A novel welded photon absorber with a total length of 600 mm has been successfully designed and manufactured. Finite-element analysis by *ANSYS* shows that the maximum temperature, equivalent stress and strain are only 31.5%, 36.2% and 1.3%, respectively, of the corresponding thresholds. The welded photon absorbers with EBW joints will be applicable in the highest-heat-load front-end in the Shanghai Synchrotron Radiation Facility Phase-II beamline project.

## Abstract 2

A novel welded photon absorber with a total length of 600 mm has been successfully designed and manufactured, and will be applicable in the highest-heat-load front-end in the Shanghai Synchrotron Radiation Facility Phase-II beamline project.

## 1. Introduction

Shanghai Synchrotron Radiation Facility (SSRF) is an advanced third-generation synchrotron radiation light source consisting of a 150 MeV electron linear accelerator, a full energy booster and a 3.5 GeV storage ring (He & Zhao, 2014 ▸; Zhao *et al.*, 2013 ▸). Insertion devices, including undulators and wigglers, are always employed to generate high-quality synchrotron light. The power output of an insertion device is up to several tens of kilowatts and its peak power density can be larger than 100 kW mrad^−2^. The photon absorber, as a critical component of a synchrotron front-end, is an ultrahigh-vacuum unit primarily for handling the high heat load from insertion devices or bending magnets (Sakurai *et al.*, 1998 ▸; Shu & Ramanathan, 2002 ▸). High-heat-load photon absorbers are mostly made of dispersion strengthened copper or CuCrZr alloy at SSRF and many other synchrotron radiation facilities. However, due to the technical requirements of high-dimensional accuracy and the low surface roughness of photon absorbers, only short ones whose lengths are no more than 300 mm could be made by low-speed wire-cut electrical discharge machining in China, which were widely used in the SSRF Phase-I project. Joining processes for vacuum, including tungsten inert gas welding (TIG) and electron beam welding (EBW), are novel ways to make a longer photon absorber by joining two shorter ones and to reduce power density.

CuCrZr, as a precipitation hardening copper alloy with excellent thermal conductivity, strength retention, good ductility and microstructural stability at elevated temperatures, developed in recent years, is widely applied in aerospace, fusion application, synchrotron radiation *etc*. (Huang *et al.*, 2019 ▸; Shueh *et al.*, 2017 ▸; Zinkle, 2016 ▸). Compared with dispersion strengthened copper, CuCrZr alloy is much cheaper and available even in our local supply vendors in China (Jin *et al.*, 2021 ▸; Sheng *et al.*, 2016 ▸). Extensive studies on the mechanical properties of CuCrZr alloy have been conducted (DeGroh *et al.*, 2008 ▸; Edwards *et al.*, 2007 ▸; Nomura *et al.*, 2002 ▸). Performances of the joints of CuCrZr alloy to other alloys have also been studied by various groups (Gillia *et al.*, 2009 ▸; Sahlot *et al.*, 2018 ▸; Wei *et al.*, 2018 ▸).

In this paper, to meet the project requirements, CuCrZr was chosen to make tensile specimens by TIG and EBW. The mechanical properties of the vacuum joints of CuCrZr to the same material at 20°C, 100°C, 200°C, 300°C and 400°C were obtained by tensile tests. An engineering conservative acceptance criteria of the vacuum joints is created by the polynomial fitting method. A novel high-heat-load photon absorber with a total length of 600 mm has been successfully designed and manufactured. Finite-element analysis by *ANSYS* shows that the maximum temperature, equivalent stress and strain on the EBW joint of the photon absorber are much lower than the corresponding thresholds, which illustrates that welded photon absorbers will be applicable in the highest-heat-load front-end in the SSRF Phase-II beamline project.

## 2. Experimental procedures

### 2.1. Material

CuCrZr is a precipitation hardened alloy with about 0.5–1.5% chrome and 0.05–0.25% zirconium. Glidcop^®^AL-15, a kind of Al_2_O_3_ dispersion strengthened copper produced by North American Höganäs High Alloys LLC in the USA, was utilized in the SSRF Phase-I front-end. Table 1 ▸ shows the physical properties of CuCrZr and Glidcop^®^AL-15 at 20°C. The mechanical properties of CuCrZr and Glidcop^®^AL-15 at different temperatures are shown in Table 2 ▸ (DeGroh *et al.*, 2008 ▸; Glidcop^®^, 2021 ▸). Compared with Glidcop^®^AL-15, the elongation of CuCrZr is worse at some temperatures, while the tensile stress and yield stress of CuCrZr are much better at all listed temperatures. In this paper, CuCrZr was chosen to make tensile specimens.

### 2.2. Welding technology

TIG welding is the most versatile welding process employed in vacuum fields where a high degree of quality and accuracy is required (Manohar *et al.*, 2018 ▸). A welding speed of 50 mm min^−1^ and a gas flow of 9 L min^−1^ were chosen to join two pieces of CuCrZr specimens using an WSE-500 argon arc welder. The test welding is done by keeping voltage at about 15 V and current at about 160 A throughout the welding process.

EBW is a fusion welding process under vacuum as it is of high energy density, low heat input and rapid cooling rate (Zeng, 1993 ▸). An EBW700 electron beam welder was employed in the experiment and pumped to a vacuum degree of 4.5 × 10^−3^ Pa. A voltage of 150 kV and a beam current of 9 mA were precisely controlled to produce a beam of high-velocity electrons. With a welding speed of 10 mm s^−1^, two pieces of CuCrZr specimens melted and flowed together on the joint.

### 2.3. Testing procedures

**Figure 1:**
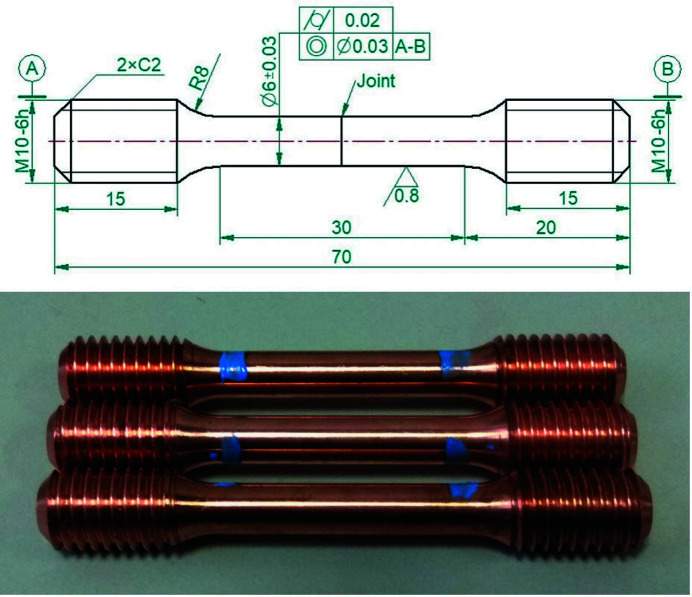
Tensile specimen (units: mm).

After welding, tensile specimens were machined according to GB/T 2651-2008 (ISO 4136: 2001, IDT), as shown in Fig. 1 ▸. Tensile tests at room temperature were conducted using a Zwick Z100TEW electronic universal testing machine based on GB/T 228.1-2010 (ISO 6892-1: 2009, MOD); a Zwick Z250 electronic universal testing machine was used at elevated temperatures adopting GB/T 4338-2006 (ISO 783: 1999, MOD).

## 3. Results and discussions

### 3.1. Testing results of TIG joints

Thirty TIG tensile specimens were separated into five groups randomly and mechanical properties at five different temperatures were obtained. The results are shown in Tables 3 ▸, 4 ▸ and 5 ▸, where *A* is elongation after fracture, 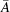 is the mean of *A*, 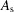 = 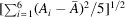 (*i* = 1–6) is the standard deviation of *A* by the Bessel formula; *R*_m_ is the tensile strength, 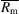 is the mean of *R*_m_, *R*_ms_ is the standard deviation of *R*_m_; *R*_p_ is the yield strength, 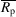 is the mean of *R*_p_, and *R*_ps_ is the standard deviation of *R*_p_. Fracture surfaces of all specimens occurred on the joints.

All TIG joints have yielding processes with poor consistency, demonstrating that TIG joints have poor ductility. From 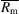 and 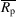 , tensile strength and yield strength decline with the rising of temperature. Meanwhile, tensile strengths and yield strengths vary a lot at the same temperature. Take the tensile strength at 400°C, for example: the maximum is nearly four times the minimum, and the biggest standard deviation is 38.2 MPa, which is even larger than half of 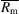 at 400°C. It is indicated that the TIG joints by the welding process have low stability and are not recommended to be applied in those cases requiring high strengths.

### 3.2. Testing results of EBW joints

Similar to Section 3.1, mechanical properties of 30 EBW tensile specimens at five different temperatures are acquired. The results are shown in Tables 6 ▸, 7 ▸ and 8 ▸. Fracture surfaces of all specimens also occurred on the joints.

All EBW joints have yielding with good consistency, demonstrating that EBW joints have better ductility. Tensile strength and yield strength decline as temperature is increased. Meanwhile, tensile strengths and yield strengths vary a little at the same temperature with a maximum difference of 46 MPa and all standard deviations are less than 20 MPa, indicating that the EBW joints by the welding process have high stability and will be preferred in practical applications.

### 3.3. Welded joints versus bulk CuCrZr

**Figure 2:**
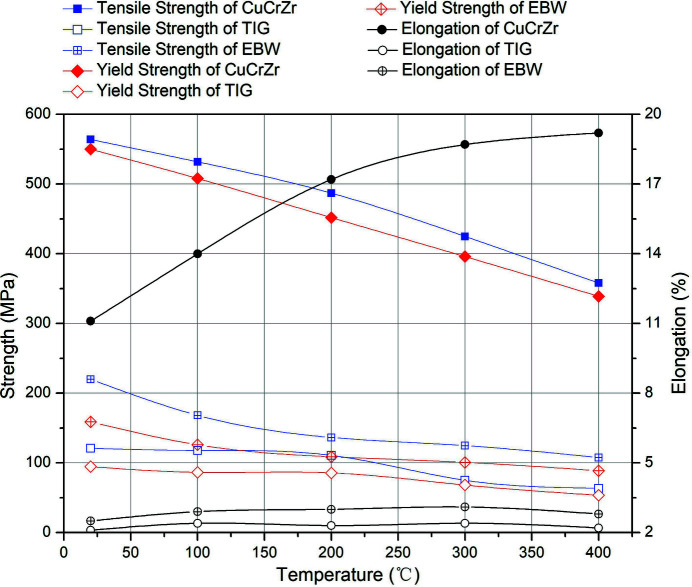
Welded joints versus bulk CuCrZr.

Mechanical properties of both welded joints are much lower than that of bulk CuCrZr, as shown in Fig. 2 ▸. The tensile strength of TIG joints is about 1/5 of the base material and 1/3 for EBW joints. The yield strength of TIG and EBW joints is only 1/6 and 1/4 of the base material, respectively. Elongation of EBW joints is approximately 1/5 of CuCrZr, while that of TIG joints is about 1/8. Changing trends of tensile strength and yield strength of both joints are consistent with that of the base material.

### 3.4. Acceptance criteria

To ensure that the high-heat-load photon absorbers can work safely during a long service period, the failure of vacuum joints due to the excessive thermal stress and strain should be avoided. It is necessary to establish the acceptance criteria when performing finite-element analysis. On the basis of the engineering conservativeness, the temperature on the joints should be no more than 250°C; thermal stress and strain should be no more than the yield strength and elongation at the temperature (Thomas *et al.*, 2016 ▸; Zhang *et al.*, 2002 ▸).

From the above testing results, formulas of yield strength and elongation based on the temperature can be obtained by the polynomial fitting method. For TIG joints, that is

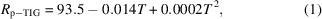




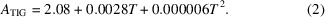

For EBW joints, that is

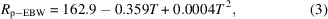




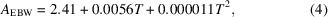

where *T* (°C) is the temperature on the joints and should be no more than 250°C. *R*_p–TIG_ (MPa) is the yield strength of TIG joints on *T*, *R*_p–EBW_ (MPa) is the yield strength of EBW joints on *T*, and *A*_TIG_ and *A*_EBW_ (%) are the elongation of the TIG joints and EBW joints at *T*, respectively.

The temperature, thermal stress and strain thresholds corresponding to both vacuum joints are listed in Table 9 ▸.

## 4. Applications

**Figure 3:**
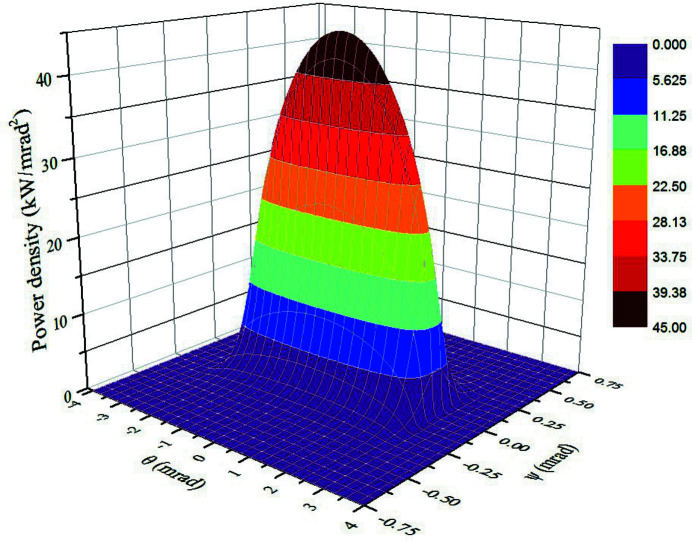
Power density distribution of the superconducting wiggler.

A superconducting wiggler, as source of the ultra-hard X-ray applications beamline (BL12SW) in the SSRF Phase-II beamline project, will generate high-energy X-rays up to 150 keV and significantly strengthen the application potential of SSRF. The total radiant heat power received by the front-end of BL12SW is over 44.7 kW, and the peak power density is approximately 45 kW mrad^−2^, which is currently the highest heat load for a front-end at SSRF. The power density distribution of the superconducting wiggler is shown in Fig. 3 ▸.

**Figure 4:**
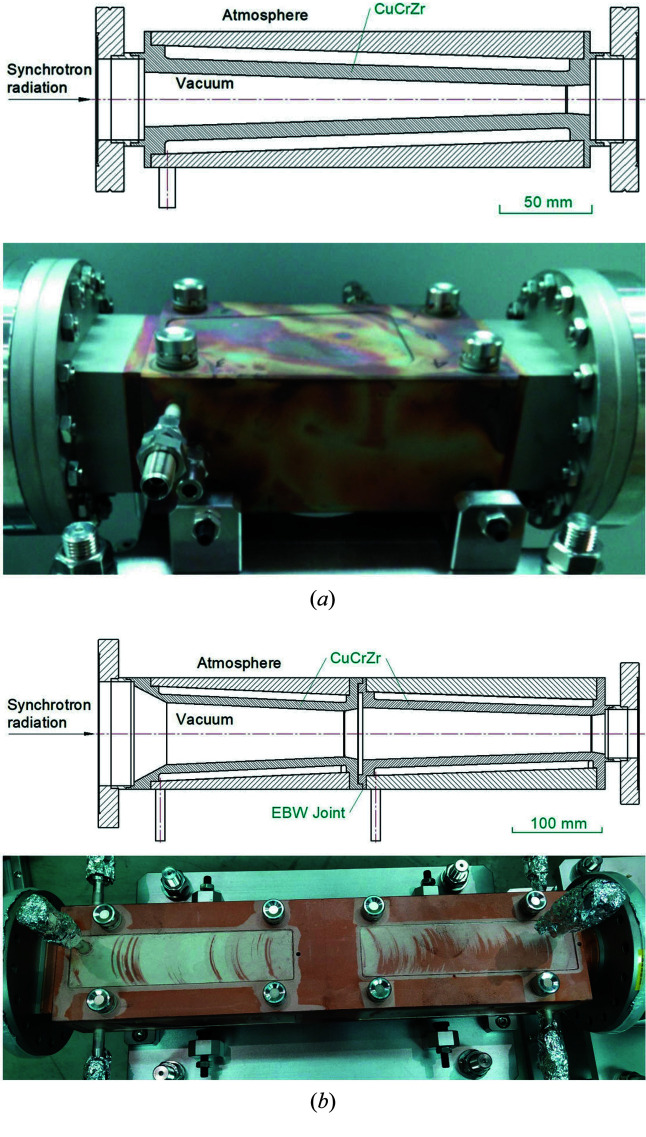
Usual photon absorber (*a*) and welded photon absorber (*b*).

Four photon absorbers made of Glidcop^®^AL-15 or CuCrZr are located in sequence to handle this radiation. Fig. 4 ▸(*a*) shows a photon absorber that was utilized in the SSRF Phase-I front-end and has only one absorber with a total length of about 300 mm. The novel photon absorber has two sub-absorbers welded to each other by EBW and a total length of up to 600 mm, as shown in Fig. 4 ▸(*b*). The inside of all the photon absorbers is in ultrahigh-vacuum while the outside is at atmospheric pressure.

**Figure 5:**
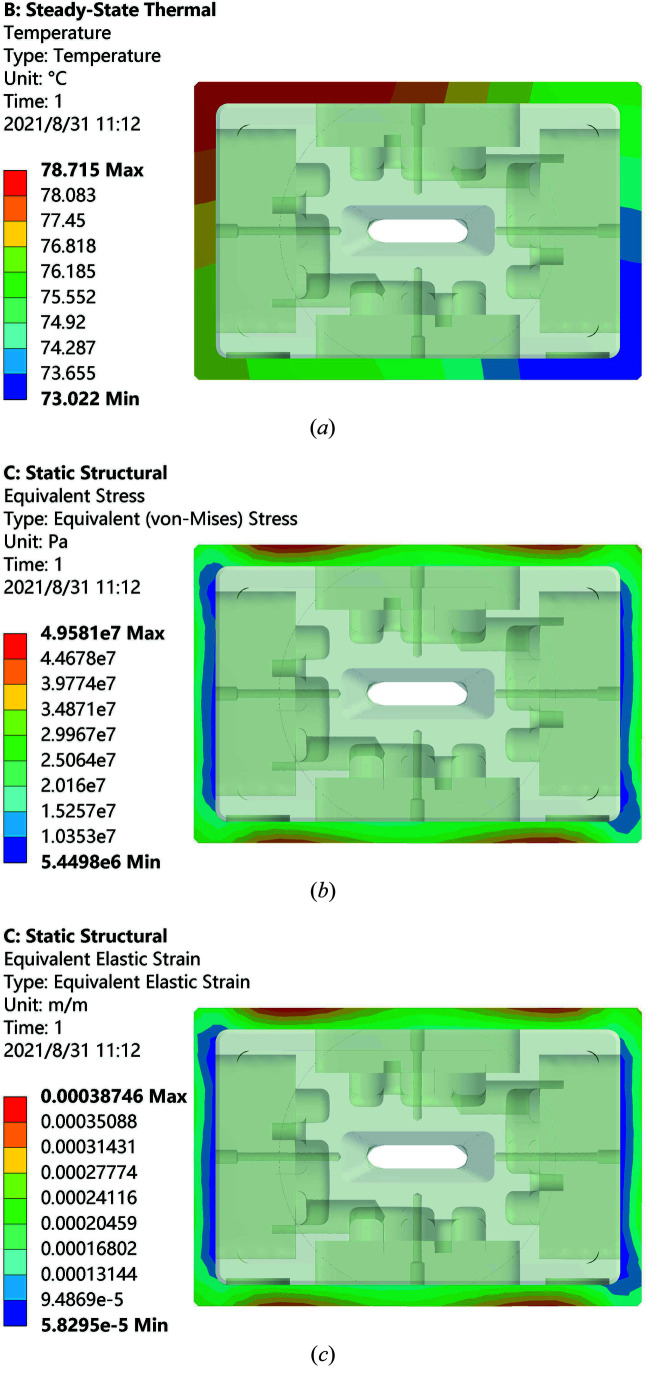
(*a*) Temperature, (*b*) equivalent stress and (*c*) equivalent strain distributions of the joint.

Finite-element analysis by *ANSYS* (Jin *et al.*, 2021 ▸; Xu *et al.*, 2015 ▸) indicates that the maximum temperature on the joint is 78.7°C, maximum equivalent stress and strain are, respectively, 49.58 MPa and 0.039%, as shown in Fig. 5 ▸. The yield strength and elongation of the EBW joint at 78.7°C can be calculated from equations (3) and (4) to be 137.1 MPa and 2.92%, respectively. Therefore, the practical maximum temperature, equivalent stress and strain are only 31.5%, 36.2% and 1.3%, respectively, of the corresponding thresholds, which indicates that the EBW joints will work safely in the highest-heat-load front-end in the SSRF Phase-II beamline project.

## 5. Conclusions

Mechanical properties of TIG joints and EBW joints of CuCrZr to the same material, which is utilized in the SSRF front-end, are obtained by tensile tests at 20°C, 100°C, 200°C, 300°C and 400°C. Testing results show that, in comparison with the base material, tensile strength and yield strength of both vacuum joints decline by 2/3 or 4/5 and elongation falls by 4/5 or more. Compared with TIG joints, EBW joints have higher strength, better ductility and more stable performance.

To ensure that the high-heat-load photon absorbers can work safely during a long service period, an engineering conservative acceptance criteria of vacuum joints is created by the polynomial fitting method. A novel welded photon absorber with a total length of 600 mm has been successfully designed and manufactured. Finite-element analysis by *ANSYS* shows that the maximum temperature, equivalent stress and strain are only 31.5%, 36.2% and 1.3%, respectively, of the corresponding thresholds.

In practical engineering applications, vacuum joints are subjected to low temperature, low stress and strain as are being kept out of direct high-power synchrotron radiation. Therefore, EBW joints of CuCrZr entirely satisfy the practical requirements and will be applicable in the highest-heat-load front-end in the SSRF Phase-II beamline project.

## Figures and Tables

**Table 1:** Physical properties of CuCrZr and Glidcop^®^AL-15

Material	Melting point (°C)	Density (g cm^−3^)	Elastic modulus (GPa)	Poisson’s ratio	Thermal conductivity (W m^−1^ °C^−1^)	Thermal expansion coefficient (°C^−1^), 20°C
CuCrZr	1075	8.9	128	0.33	330	1.70 × 10^−5^
Glidcop^®^AL-15	1083	8.9	130	0.33	365	1.66 × 10^−5^

**Table 2:** Mechanical properties of CuCrZr and Glidcop^®^AL-15

Material	Mechanical properties	20°C	100°C	200°C	300°C	400°C
CuCrZr	Tensile stress (MPa)	564	532	487	425	358
	Yield stress (MPa)	550	508	452	396	339
	Elongation (%)	11.1	14	17.2	18.7	19.2
Glidcop^®^AL-15	Tensile stress (MPa)	420	380	330	292	263
	Yield stress (MPa)	355	332	304	277	248
	Elongation (%)	27	25	21	16.5	12

**Table 3:** Elongation of TIG joints

Temperature	*A* (%)							
	*A*_1_	*A*_2_	*A*_3_	*A*_4_	*A*_5_	*A*_6_	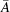	*A*_s_
20°C	1.8	2	3	2.5	1.5	1.6	2.1	0.6
100°C	1.9	2.5	2	1.6	2.6	3.5	2.4	0.7
200°C	2.5	2	1.8	3.5	2.1	1.7	2.3	0.7
300°C	1.5	3.4	2.9	2.5	2.1	2	2.4	0.7
400°C	3	1.6	2.9	1.5	2.1	2.2	2.2	0.6

**Table 4:** Tensile strength of TIG joints

Temperature	*R*_m_ (MPa)							
	*R*_m1_	*R*_m2_	*R*_m3_	*R*_m4_	*R*_m5_	*R*_m6_	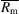	*R*_ms_
20°C	91.5	97.5	174.5	108	136	118	120.9	30.6
100°C	129	83.5	139	146	103.5	105	117.7	24.1
200°C	93.5	161	85.5	102	118	105	110.8	26.9
300°C	73.5	89.5	76	49	86	76.5	75.1	14.2
400°C	32.5	52.5	36.5	93	127	39.5	63.5	38.2

**Table 5:** Yield strength of TIG joints

Temperature	*R*_p_ (MPa)							
	*R*_p1_	*R*_p2_	*R*_p3_	*R*_p4_	*R*_p5_	*R*_p6_	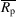	*R*_ps_
20°C	87.5	82	113.5	95	101	88	94.5	11.4
100°C	75	75	106	87	97	77.5	86.3	12.9
200°C	62.5	121.5	83	68	90	89	85.7	20.8
300°C	70	80.5	55.5	43.5	85.5	74.5	68.3	15.9
400°C	31	49.5	36	76	94	34.5	53.5	25.8

**Table 6:** Elongation of EBW joints

Temperature	*A* (%)							
	*A*_7_	*A*_8_	*A*_9_	*A*_10_	*A*_11_	*A*_12_	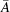	*A*_s_
20°C	2.1	2.5	2.4	3	2.6	2.5	2.5	0.3
100°C	3.4	3	2.5	2.6	3.1	2.5	2.9	0.4
200°C	2.8	3.5	3	2.5	2.7	3.6	3.0	0.4
300°C	2.7	3	3.1	3.5	3	3.3	3.1	0.3
400°C	2.5	2.4	2.9	3.4	3.1	2.6	2.8	0.4

**Table 7:** Tensile strength of EBW joints

Temperature	*R*_m_ (MPa)							
	*R*_m7_	*R*_m8_	*R*_m9_	*R*_m10_	*R*_m11_	*R*_m12_	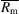	*R*_ms_
20°C	199	201	219	235	223	243	220	17.7
100°C	142	178	184	172	188	145	168.2	19.9
200°C	155	139	141	128	129	127	136.5	10.8
300°C	123	136	118	119	135	118	124.8	8.5
400°C	131	106	88	119	98	104	107.7	15.3

**Table 8:** Yield strength of EBW joints

Temperature	*R*_p_ (MPa)							
	*R*_p7_	*R*_p8_	*R*_p9_	*R*_p10_	*R*_p11_	*R*_p12_	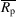	*R*_ps_
20°C	176	156	149	153	152	168	159	10.6
100°C	125	108	149	115	140	117	126	15.8
200°C	125	106	86	98	122	115	108.7	15
300°C	98	88	91	102	122	103	100.7	12.0
400°C	90	94	76	97	85	90	88.7	7.4

**Table 9:** Engineering conservative acceptance criteria of vacuum joints

	TIG joint	EBW joint
Maximum temperature (°C)	≤250	
Maximum equivalent stress (MPa)	≤93.5 − 0.014*T* + 0.0002*T* ^2^	≤162.9 − 0.359*T* + 0.0004*T* ^2^
Maximum equivalent strain (%)	≤2.08 + 0.0028*T* + 0.000006*T* ^2^	≤2.41 + 0.0056*T* + 0.000011*T* ^2^
